# Development of a Predictive Model for the Progression of Subjective Cognitive Decline: A Longitudinal Study

**DOI:** 10.1002/brb3.70719

**Published:** 2025-08-12

**Authors:** Wenyi Li, Jiwei Jiang, Qiwei Ren, Min Zhao, Linlin Wang, Shiyi Yang, Shirui Jiang, Tianlin Jiang, Huiying Zhang, Jun Xu

**Affiliations:** ^1^ Department of Neurology, Beijing Tiantan Hospital Capital Medical University Beijing China; ^2^ China National Clinical Research Center For Neurological Diseases, Beijing Tiantan Hospital Capital Medical University Beijing China; ^3^ Health Management Center, Beijing Tiantan Hospital Capital Medical University Beijing China

**Keywords:** Alzheimer's disease, neuroimaging, prediction model, risk factors, subjective cognitive decline

## Abstract

**Background:**

Subjective cognitive decline (SCD) is a preclinical stage of Alzheimer's disease (AD). However, the factors influencing SCD progression remain unclear. It is necessary to develop a model for predicting cognitive progression in SCD.

**Methods:**

96 participants with SCD and 36 healthy controls (HCs) were enrolled from the Chinese Imaging, Biomarkers, and Lifestyle study between January 1 and June 30, 2022. Of these, 70 completed approximately 12 months of follow‐up visits. Clinical, cognitive assessment, and neuroimaging data were collected. Cox proportional‐hazard regression models were used to investigate the risk factors and construct a nomogram.

**Results:**

Compared to HCs, participants with SCD had higher Pittsburgh Sleep Quality Index (PSQI) scores, indicating they had poorer sleep quality, and had higher cerebral blood flow (CBF) in bilateral hippocampus, thalamus, and left precuneus (all *p* < 0.05). Poorer sleep quality and left precuneus CBF were independently associated with SCD progression (all *p* < 0.05). The nomogram constructed with these factors achieved good discriminative ability, with an AUC of 0.785 (95% CI: 0.609–0.960) and a coherence index of 0.840 (95% CI: 0.733–0.948). The calibration curves showed significant agreement between the model and actual observations, and the decision curve analysis of the model showed clinical benefit.

**Conclusions:**

A predictive model for SCD progression constructed based on risk factors including PSQI scores and left precuneus CBF showed good accuracy and discrimination ability, and it may provide valuable insights for early stage screening of AD.

## Graphical abstract

This longitudinal study enrolled healthy controls and subjective cognitive decline (SCD) and collected their multi‐modal data, which indicated that sleep quality and cerebral blood flow on left precuneus at baseline were related to SCD progression. We developed a predictive model that presented good discriminative ability and clinical benefit.

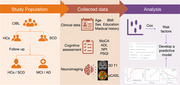

## 1 Introduction

With a rapidly increasing global aging population, Alzheimer's disease (AD) and related cognitive impairment have emerged as a serious public health threat (2024 Alzheimer's disease facts and figures 2024). The disease usually progresses gradually from subjective cognitive decline (SCD) to mild cognitive impairment (MCI) and then to dementia. The concept of preclinical AD and early intervention has sparked a renewed interest in SCD, which is characterized by persistent self‐reported cognitive decline (lasting ≥6 months) while demonstrating preserved performance on age‐adjusted standardized neuropsychological assessments (Jessen et al. 2020). At the stage of MCI, patients exhibit objective evidence of mild deficits in cognitive function, with neuropsychological assessment sores that are 1–1.5 standard deviations (SD) below those normal for their adjusted age and education, while their daily living activities remain largely preserved (Petersen 2004). The condition eventually progresses to dementia, which is characterized by impairments in two or more cognitive domains (typically >1.5 SD below age‐ and education‐adjusted norms on neuropsychological assessments), accompanied by a decline in daily living activities (Albert et al. 2011). A longitudinal study reported that SCD occurred approximately 10 years before the diagnosis of dementia (Cappa et al. 2024). Recent studies have shown that elderly individuals with SCD have an approximately 30% conversion rate to MCI or dementia and present more pathological changes related to AD than those without (An et al. 2024; Jessen et al. 2023). A recent epidemiological study reported that the prevalence of SCD in China was approximately 46% (Xue et al. 2023). Therefore, identifying the factors influencing SCD and providing early predictive clues are crucial to achieve preclinical screening and provide targeted interventions.

However, traditional diagnostic tools, such as lumbar puncture and positron emission tomography, are expensive and invasive, making them difficult to perform in the early stages of dementia. With the development of magnetic resonance imaging (MRI) technology, multimodal MRI, including structural MRI and functional MRI, has been widely used for screening and follow‐up of neurodegenerative diseases (Chouliaras and O'Brien 2023). Previous studies have found that structural MRI is widely employed in AD detection and early prediction, leveraging its sensitivity to gray matter atrophy and cortical thinning attributed to the deposition of amyloid‐β (Aβ) (Dolci et al. 2025). Prior evidence suggests that individuals with SCD presenting AD‐like atrophy patterns exhibit higher rates of progression to MCI/AD (Karakasli et al. 2024; Lerch et al. 2024). Evidence also indicates that AD is frequently accompanied by neurovascular pathology, and the accumulation of Aβ has been shown to induce cellular dysfunction, thereby contributing to vascular impairment (Chaudhuri et al. 2025). Arterial spin labeling (ASL) is a noninvasive cerebral perfusion MRI technique that may serve as a prospective cerebral blood flow (CBF) biomarker for the diagnosis and monitoring of AD (Meng et al. 2023). Recent studies have shown that the ASL‐based quantification of CBF can detect early perfusion change in preclinical AD, even prior to the onset of structural changes (Guo et al. 2024). Moreover, hypoperfusion in AD‐vulnerable regions, including the posterior cingulate cortex and precuneus, has been associated with Aβ accumulation and the progression from SCD to MCI (Thropp et al. 2024; Taghvaei et al. 2025).

SCD may be the earliest clinical manifestation of AD and an important warning sign of cognitive decline. This stage provides an important window of opportunity to delay or even to prevent progression to AD. Due to the complex etiology of SCD, it is difficult for clinicians to make the accurate and timely identification of high‐risk SCD. Therefore, in this study, we aimed to analyze the clinical characteristics, neuropsychological assessment, and neuroimaging features (including the brain structure and CBF) of SCD to identify the risk factors of progression and then develop a predictive model to provide a reliable method for early screening and intervention.

## 2 Methods

### 2.1 Dataset and Study Design

In this longitudinal study, we enrolled 132 participants with non‐objective cognitive impairment from the Chinese Imaging, Biomarkers, and Lifestyle (CIBL) Study between January 1, 2021, and June 30, 2022. The participants comprised 96 participants with SCD and 36 healthy controls (HCs). A total of 47 participants with SCD and 17 HCs completed a median follow‐up period of 12 months (interquartile range: 11–13 months), with durations ranging from a minimum of 6 months to a maximum of 24 months. The characteristics of participants and the procedures of the CIBL study have been described elsewhere (Jiang et al. 2023; Jiang et al. 2024). All participants provided written consent prior to participation in the study. The CIBL study was approved by the Institutional Review Board of the Beijing Tiantan Hospital of Capital Medical University (KY‐2021‐028‐01) and was registered at Chictr.org.cn (ChiCTR2100049131).

The inclusion criteria for participants with SCD at baseline were based on the framework proposed by the SCD International Working Group in 2020: (1) Age at enrollment of 60 years or older; (2) the individual complained of a persistent decline in memory rather than other cognitive domains over the past 5 years and expressed concern about the cognitive decline; (3) cognitive test scores within the normal range after adjusting for age and years of education; (4) Subjective Cognitive Decline Questionnaire 9 (SCD‐Q9) scores exceeding five points; and (5) right‐handedness (Jessen et al. 2020; Hao et al. 2017).

The inclusion criteria for HCs were as follows: (1) Age at enrollment of 60 years or older; (2) no cognitive decline noted; (3) cognitive test scores within the normal range after adjusting for age and years of education; (4) SCD‐Q9 scores less than five points; and (5) right‐handedness.

The following exclusion criteria were applied: (1) met the diagnostic criteria for AD proposed by the 2018 or 2011 NIA‐AA or those for MCI proposed by Petersen (Petersen 2004; Albert et al. 2011; Jack et al. 2018); (2) considered to have other central nervous system disorders besides AD that may lead to cognitive dysfunction, such as frontotemporal lobe dementia, dementia with Lewy bodies, Parkinson's disease, and vascular cognitive impairment; (3) mental disorders defined by Diagnostic and Statistical Manual of Mental Disorders, fifth Edition (DSM‐5); (4) alcohol abuse, drug addiction, poisoning, or exposure to harmful substances such as radiation; (5) claustrophobia or the presence of contraindications to 3.0T MRI, such as metal implants, pacemakers, and dental implants, as well as poor image quality or loss of signal, which prevents the completion of subsequent data processing; (6) taken sedatives, caffeine, nicotine, or other drugs that may affect the CBF, within 1 day before examination; and (7) unstable vital signs.

The study selection and analysis procedures are shown in Figure S1.

### 2.2 Demographic Data Collection and Neuropsychological Assessment

We collected data on age of enrollment, sex, years of education, body mass index (BMI), and medical history, including hypertension, diabetes, hyperlipidemia, stroke/transient ischemic attack (TIA), smoking, drinking, and family history of dementia, using the standardized enrollment process.

Global cognitive function was assessed using the Montreal Cognitive Assessment (MoCA). The self‐care and tool‐use capabilities were evaluated using the Activities of Daily Living (ADL) scale, neuropsychiatric symptoms were assessed using the Neuropsychiatric Inventory (NPI), and sleep quality was assessed using the Pittsburgh Sleep Quality Index (PSQI) (Supporting Information Materials 1) (Taghvaei et al. 2025; Jiang et al. 2023; Islam et al. 2023; Jiang et al. 2025). All assessments were conducted by a professional neurologist in a quiet assessment room.

### 2.3 *APOE* Genotype

We collected elbow venous blood when the subjects were fasting in the morning. The *APOE* genotype was determined based on allelic combinations of the single‐nucleotide polymorphisms (SNPs) rs7412 and rs429358 using the Illumina WeGene V3 array (Illumina iScan System; Illumina, Inc., San Diego, CA, USA). According to the presence or absence of the *APOE*ɛ4 allele, we divided the participants into the *APOE*ε4 carrier group (ε2/ε4, ε3/ε4, and ε4/ε4) and non‐*APOE*ε4 carrier group (ε2/ε2, ε2/ε3, and ε3/ε3).

### 2.4 MRI Acquisition and Processing

MRI data were collected using a 3.0 Tesla magnetic resonance scanner with 48‐channel head coils (SIGNA Premier; GE Healthcare, Chicago, IL, USA). Before the MRI scan, all participants were in a resting state for a minimum of 15 min. 3D T1 images were acquired with a 3‐dimensional magnetization‐prepared rapid gradient‐echo sequence with the following parameters: Repetition time (TR) = 7.3 ms; flip angle = 12°; field of view = 256 × 256 mm^2^; acquisition matrix = 256 × 256; slice thickness = 1.0 mm; slice number = 176; and scan time = 4 min 56 s. For multi‐delay pCASL, the imaging parameters were as follows: TR = 9315 ms, echo time = 11.2 ms, field of view = 220 × 220 mm, slice thickness = 3.0 mm, slice number = 48, acquisition matrix = 512 × 512, and post‐labeling delay = 1000, 1361, 1739, 2141, 2577, 3067, and 3658 ms (Lindner et al. 2023).

The imaging data were processed using CereFlow software (Anying Technology [Beijing] Co., Ltd., China), with the following steps: (1) The reconstruction of CBF images was achieved by uploading 3D T1 and original ASL images to the CereFlow platform; (2) the M0 image (proton density map of ASL) was co‐registered with the 3D T1 image; (3) the T1 imaging was normalized to the Montreal Neurological Institute (MNI) template (Rolls et al. 2020), and the CBF image was normalized to MNI template space using spatial transformation matrix; (4) the Automated Anatomical Labeling Atlas 3 (AAL3) was employed to delineate cerebral regions and extract the volume and CBF value of the region of interest (ROI) (J. Li et al. 2023). In this study, brain regions associated with cognition, including the bilateral caudate nucleus, lentiform nucleus, insula, hippocampus, thalamus, amygdala, precuneus, and middle temporal gyrus, were selected as ROIs for analysis (Ding et al. 2023; Goschel et al. 2023).

### 2.5 Longitudinal Analysis

Participants completed a face‐to‐face follow‐up visit after approximately 12 months. The data collected at follow‐up included clinical characteristics, neuropsychological assessment, and neuroimaging. The primary outcome used to define progression was based on clinical diagnostic criteria. MCI diagnosis was determined according to the Petersen criteria, which consist of (1) objective cognitive impairment in one or more domains (MoCA score ≤ 24 for individuals with > 6 years of education, ≤ 20 for those with 1–6 years of education, and ≤ 14 for those with no formal education); (2) preserved independence in functional activities (ADL score = 20); and (3) absence of dementia (Petersen 2004). AD diagnosis was based on the 2011 or 2018 NIA‐AA criteria, requiring evidence of progressive cognitive decline affecting daily function and meeting established clinical thresholds for dementia (ADL >20) (Albert et al. 2011; Jack et al. 2018).

### 2.6 Statistical Analysis

IBM SPSS 29.0 (v29.0; IBM Corp., Armonk, NY, USA) statistical software and R software (version 4.1.2) were used to analyze the data. Continuous variables with a normal distribution were described using the mean and SD, whereas continuous variables with a non‐normal distribution were described using the median and interquartile range. An independent *t*‐test was employed for normally distributed data, whereas the Mann–Whitney *U* test was employed for non‐normally distributed data. Categorical variables were presented as total numbers (*n*) and percentages (%), and the chi‐square test or Fisher's exact test was used to assess statistical differences. Missing data were handled using pairwise deletion, which helped to maintain the integrity of the dataset and ensure the accuracy of the analysis results. Univariate and multivariate Cox proportional hazards regression models were used to identify the independent factors influencing SCD progression and develop a predictive and visual nomogram model for the comprehensive evaluation and risk stratification of SCD, thus improving the accuracy of its prognostic assessment in the clinic.

The nomogram model was developed based on the multivariate Cox proportional hazards model, incorporating independent predictors that were statistically significant in the multivariate analysis. The nomogram construction process comprised the following four steps: (1) Variable Selection: Candidate predictors were initially screened using univariate Cox regression analysis. Variables with *p* < 0.05 were subsequently entered into a multivariate Cox regression model to identify the independent predictors associated with SCD progression. (2) Model construction: A multivariate Cox regression model was then constructed based on the selected independent variables. The regression coefficients obtained from the final model were used to develop the nomogram. (3) Nomogram Visualization: The nomogram was visualized by assigning a weighted point score to each predictor according to its corresponding regression coefficient. The total point score was then mapped to an individual's predicted probability of SCD progression. The final predictive formula can be expressed as follows:

$$P\left(Y=1\right)=1/\left(1+\hat{e}\left(-\left(\beta_{0}+\beta_{1}X_{1}+\beta_{2}X_{2}+\cdots +\beta_{n}X_{n}\right)\right)\right.$$

where *P* (*Y* = 1) represents the predicted probability of the occurrence of SCD progression, *β*₀ represents the intercept, *β*₁–*β*_n_ are the regression coefficients, and *X*₁–*X*_n_ are the predictor variables. (4) Model Validation: The nomogram was internally validated using a bootstrap resampling method (1000 repetitions). Discriminative performance was evaluated using Harrell's concordance index (C‐index) and receiver operating characteristic (ROC) analysis employing the area under the curve (AUC). C‐index approaching 1 signifies robust model predictability. Model calibration was quantified through the Hosmer–Lemeshow goodness‐of‐fit test, which can reflect the agreement between predicted probabilities and observed outcomes. Clinical utility was further examined via decision curve analysis (DCA) to determine the net benefit of the nomogram across risk thresholds (Wang et al. 2024). The test level was bilateral, and *p * < 0.05 was considered statistically significant.

## 3 Results

### 3.1 Baseline Clinical Characteristics of Both Groups

A total of 132 participants were included, and the average enrollment age of the study population was (62.07 ± 7.86) years. Female participants accounted for 75%, and *APOE*ε4 carriers accounted for 17.42% of the study population. Compared with the HCs, participants with SCD had higher PSQI scores, which indicate poorer sleep quality (*p* = 0.002). The baseline clinical characteristics of the HCs and participants with SCD are presented in Table 1.

**TABLE 1:** Baseline characteristics of clinical in HCs and participants with SCD.

	HC (*n* = 36)	SCD (*n* = 96)	*t*/*χ* ^2^/*z*	*p* value
Age of enrollment (mean ± SD, years)	63.14 ± 8.89	61.63 ± 7.41	0.988	0.325
Sex (female, [%])	23 (63.89)	76 (79.17)	3.259	0.071
Education (median [IQR], years)	15.00 (13.00,16.00)	13.00 (11.00,16.00)	−0.647	0.518
*APOE*ε4 carrier (yes, *n* [%])	3 (8.33)	20 (20.83)	—	0.123
BMI (mean ± SD, kg/m^2^)	24.57 ± 2.70	23.89 ± 2.72	0.198	0.196
Hypertension (yes, *n* [%])	10 (27.78)	35 (36.50)	0.878	0.349
Diabetes (yes, *n* [%])	3 (8.33)	16 (16.67)	—	0.277
Hyperlipidemia (yes, *n* [%])	12 (33.33)	38 (39.58)	0.435	0.510
Stroke/TIA (yes, *n* [%])	2 (5.56)	7 (7.29)	—	0.997
Smoking (yes, *n* [%])	8 (22.22)	18 (18.75)	0.200	0.655
Drinking (yes, *n* [%])	6 (16.67)	7 (7.29)	1.377	0.241
Family history of dementia (yes, *n* [%])	8 (22.22)	26 (27.08)	0.468	0.494
MoCA (median [IQR], scores)	27.00 (26.50, 28.00)	26.50 (26.00, 27.00)	−0.604	0.546
ADL (median [IQR], scores)	20.00 (20.00, 20.00)	20.00 (20.00, 20.00)	0.004	0.999
NPI (median [IQR], scores)	0.00 (0.00, 0.50)	0.00 (0.00, 2.00)	−1.662	0.096
PSQI (median [IQR], scores)	3.00 (2.00, 7.50)	7.00 (5.00, 12.00)	−3.154	0.002

*Note*: —: *p* values were evaluated using Fisher exact tests.

Abbreviations: ADL, Activities of Daily Living; APOEε4, apolipoprotein E epsilon 4; BMI, body mass index; HC, healthy control; MoCA, Montreal cognitive assessment; NPI, Neuropsychiatric Inventory; PSQI, Pittsburgh Sleep Quality Index; SCD, subjective cognitive decline.

### 3.2 Baseline Cerebral Regional Volume and CBF Value of Both Groups

The baseline regional volume and CBF value of the HCs and participants with SCD are presented in Table 2. Participants with SCD had a higher CBF in the bilateral hippocampus, bilateral thalamus, and left precuneus than the HCs (all *p* < 0.05), and there was no significant difference between the two groups in terms of the CBF of the bilateral caudate, bilateral insula, bilateral amygdala, bilateral MTG, right precuneus, and volume of all the ROIs (all *p* > 0.05).

**TABLE 2:** Baseline regional volume CBF in HCs and patients with SCD.

ROIs	Volume (mL)				CBF (mL/100 g/min)			
	HC (*n* = 36)	SCD (*n* = 96)	*t* value	*p* value	HC (*n* = 36)	SCD (*n* = 95)	*t* value	*p* value
Caudate nucleus_L	5.61 ± 0.63	5.70 ± 0.87	−0.544	0.588	33.44 ± 10.51	34.85 ± 8.56	−0.795	0.428
Caudate nucleus_R	5.90 ± 0.77	5.95 ± 0.89	−0.273	0.785	36.26 ± 9.41	36.75 ± 7.53	−0.312	0.756
Insula_L	11.98 ± 1.20	12.38 ± 1.32	−1.587	0.115	50.58 ± 13.10	52.10 ± 11.15	−0.710	0.479
Insula_R	11.82 ± 1.09	12.14 ± 1.17	−1.408	0.161	54.96 ± 14.80	55.42 ± 10.79	−0.197	0.844
Hippocampus_L	7.04 ± 0.94	7.07 ± 0.93	−0.188	0.851	42.98 ± 11.51	47.49 ± 9.50	−2.291	0.024
Hippocampus_R	7.01 ± 0.93	6.84 ± 0.78	1.068	0.288	41.57 ± 12.10	46.77 ± 9.27	−2.633	0.009
Thalamus_L	7.60 ± 0.89	7.65 ± 1.10	−0.245	0.807	42.30 ± 13.24	47.61 ± 10.01	−2.474	0.015
Thalamus_R	6.98 ± 0.87	7.06 ± 1.00	−0.397	0.692	42.53 ± 12.60	47.20 ± 9.34	−2.315	0.022
Amygdala_L	1.66 ± 0.33	1.74 ± 0.29	−1.328	0.187	42.45 ± 11.83	42.70 ± 9.32	−0.124	0.901
Amygdala_R	1.92 ± 0.31	2.00 ± 0.30	−1.295	0.099	40.18 ± 12.42	40.59 ± 8.46	−0.216	0.829
Precuneus_L	16.60 ± 2.17	16.85 ± 1.84	−0.673	0.520	48.80 ± 18.93	57.23 ± 13.51	−2.874	0.005
Precuneus_R	19.15 ± 2.17	19.44 ± 1.97	−0.737	0.462	49.84 ± 17.76	55.16 ± 12.89	−1.895	0.060
MTG_L	29.04 ± 3.39	29.06 ± 2.80	−0.024	0.981	54.33 ± 17.18	56.95 ± 12.50	−0.883	0.336
MTG_R	29.99 ± 3.74	30.07 ± 2.76	−0.132	0.896	52.27 ± 17.58	54.89 ± 12.39	−0.885	0.339
Whole brain	578.25 ± 42.28	577.14 ± 39.30	0.141	0.888	47.07 ± 14.78	50.12 ± 11.05	−1.241	0.220

Abbreviations: CBF, cerebral blood flow; HC, healthy control; MTG, middle temporal gyrus; SCD, subjective cognitive decline.

### 3.3 Univariate Cox Proportional Hazard Regression Model for SCD Progression

Seventy participants who completed a median follow‐up period of 12 months (interquartile range: 11–13 months) were included in the longitudinal analysis. There were no differences in baseline characters between participants who completed follow‐up and those who were lost to follow‐up (Table S1). A total of 21 participants had progressed to MCI, of which 19 were from the SCD group and two from the HC group. Compared with HCs, participants with SCD at baseline had a higher rate of progression to MCI (*p* = 0.045, Table S2).

**FIGURE 1:**
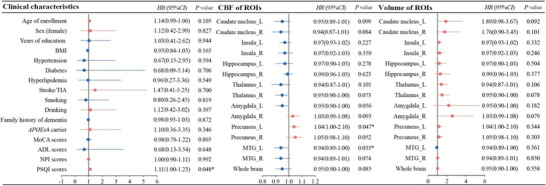
Univariate Cox proportional‐hazard regression models for progression to MCI in patients with SCD. ADL, activities of daily living; *APOE*ε4, apolipoprotein E epsilon 4; BMI, body mass index; CBF, cerebral blood flow; CI, confidence interval; HR, hazard ratio; MCI, mild cognitive impairment; MoCA, Montreal cognitive assessment; MTG, middle temporal gyrus; NPI, Neuropsychiatric Inventory; PSQI, Pittsburgh Sleep Quality Index.

Risk factors for SCD progression were analyzed using univariate and multivariate Cox proportional hazards regression models. The univariate Cox proportional hazards regression models included baseline clinical characteristics, regional CBF, and the volume of participants with SCD. The results showed that higher PSQI scores (HR = 1.11, 95% CI: 1.00–1.23, *p* = 0.048) and higher CBF of the left precuneus (HR = 1.08, 95% CI: 1.00–1.17, *p* = 0.047) were associated with a higher risk of progression to MCI. Conversely, lower CBF of the left middle temporal gyrus (HR = 0.94, 95% CI = 0.89–1.00, *p* = 0.033) was associated with a higher risk of progression to MCI. These findings are presented in Figure 1.

### 3.4 Multivariate Cox Proportional‐Hazard Regression Models for SCD Progression

Factors that were statistically significant in the univariate Cox proportional‐hazard regression models were included in the multivariate Cox proportional‐hazard regression model, while age and sex were corrected as confounders (Table 3). The results showed that higher PSQI scores (HR = 1.23, 95% CI: 1.06–1.42, *p* = 0.005) and higher CBF of left precuneus (HR = 1.05, 95% CI = 1.00–1.10, *p* = 0.041) at baseline were independent risk factors for SCD progression.

**TABLE 3:** Multivariate Cox regression model for progression in patients with SCD.

Factors	*B*	*SE*	Wald	HR	95% CI	*p* value
Age of enrollment	0.01	0.04	0.01	1.00	0.93–1.08	0.942
Sex (female)	0.26	0.62	0.18	1.30	0.39–4.33	0.672
PSQI scores	0.21	0.07	7.82	1.03	0.90–1.18	0.005
CBF of Precuneus_L	0.05	0.02	4.19	1.05	1.00–1.10	0.041
CBF of MTG_L	−0.20	0.03	0.86	0.98	0.93–1.03	0.353

Abbreviations: CBF, cerebral blood flow; CI, confidence interval; HR, hazard ratio; MTG, middle temporal gyrus; PSQI, Pittsburgh Sleep Quality Index; SE, standard error.

### 3.5 Nomogram Development and Validation

**FIGURE 2:**
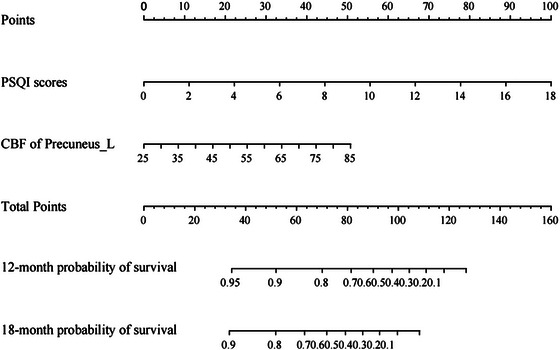
A nomogram for predicting the SCD progression. Nomogram to calculate the risk score and predict the probability of SCD progression. By drawing an upward line from the corresponding values of the two predictors to the “points” line, and the points were summed and plotted on the “Total points” line, it corresponds to prediction of SCD progression in the “risk of event” line. CBF, cerebral blood flow; PSQI, Pittsburgh Sleep Quality Index.

In our final model, PSQI scores and CBF in the left precuneus were retained as independent risk factors for progression. Each predictor was assigned a point score proportional to its regression coefficient (*β*) in the Cox model. The total points of the nomogram were calculated by assigning each predictor a score proportional to its regression coefficient. Specifically, the point assignment was based on the following formula: Total points = 1.218 × PSQI scores + CBF in the left precuneus (Figure 2).

**FIGURE 3:**
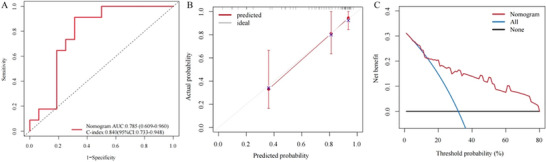
Validation of the predictive model. ROC curves of 1‐year progressive prediction in participants with SCD (A); 1‐year progressive calibration curves for participants with SCD (B); DCA for the nomogram for predicting the progression to MCI in SCD.

Accuracy and discrimination were tested using the C‐index, AUC, and calibration curves. Figure 3 shows that the AUC value of the 1‐year progressive rates of participants with SCD was 0.785 (0.609, 0.960) and the C‐index was 0.840 (95% CI: 0.733, 0.948), indicating that the model could better predict the risk of SCD progression. DCA demonstrated that this model was clinically viable.

## 4 Discussion

Compared with HCs, participants with SCD had higher PSQI scores, maenad poorer sleep quality, higher CBF in the bilateral hippocampus, thalamus, and left precuneus, and a higher rate of progression to MCI. Inferior sleep quality and increased CBF in the left precuneus were independent risk factors for progression to MCI in participants with SCD. In this longitudinal cohort study, we constructed a predictive model based on the risk factors of SCD progression, which was conducive to the early identification of participants with SCD and the comprehensive intervention and management of high‐risk groups.

The presence of SCD was associated with an increased risk of progression to MCI, which is consistent with previous studies (G. R. Cheng et al. 2023; Zlatar et al. 2022). A previous meta‐analysis demonstrated that participants with SCD were at a higher risk of dementia compared with non‐SCD counterparts. Furthermore, approximately one‐third of dementia cases are attributed to AD (Arora et al. 2024). Concurrently, SCD represents the earliest period of treatment demand, accounting for approximately 21%–29% of memory clinics. Previous randomized controlled trials have demonstrated that modifying risk factors through non‐pharmacological interventions, such as cognitive training and physical exercise during SCD, may reduce or delay the onset of dementia (Lee et al. 2024; C. H. Cheng et al. 2024). Consequently, SCD as a significant risk factor for dementia and the “golden window period” for early diagnosis and treatment have become the main target group of an increasing number of prediction‐ and intervention‐related research subjects.

Our results revealed that poorer sleep quality was an independent risk factor for SCD. A previous study demonstrated that sleep disruption inhibits glutamate, γ‐aminobutyric acid, and cyclic adenosine monophosphate. In addition, the decrease in sleep time will lead to the aggravation of AD‐related pathology, mainly through the decrease in the Aβ clearance rate of microglia and brain lymphoid system. Moreover, the prolongation of wakefulness time impairs protein homeostasis, resulting in the production of misfolded proteins, inducing neurotoxicity, and ultimately leading to the impairment of neuronal function (Parhizkar et al. 2023). In a large community cohort study, increased PSQI scores were also found to be associated with more serious subjective memory complaints, with further analysis demonstrating that poorer subjective sleep quality, longer sleep latency, and lower sleep efficiency were significantly related to the progression of SCD (Kim et al. 2021). In a randomized controlled study of SCD with poor sleep quality, it was found that the sleep efficiency and cognitive performance of participants with SCD improved after 5 days of transcranial photobio‐modulation treatment (Zhao et al. 2022). This study also suggested that it may be necessary to screen and treat sleep disorders as intervenable risk factors in participants with SCD.

In 2023, the most recent guidelines for AD proposed the “ATNIVS” diagnostic framework, which prompted the use of ASL as an imaging marker related to degenerative changes and vascular injury in the future clinical diagnosis of AD, based on original imaging markers such as structural MRI and positron emission computed tomography (Jack et al. 2024). Concurrently, ASL is advantageous in that it is noninvasive and repeatable, rendering it suitable for screening in the early stages of SCD and follow‐up monitoring.

Previous studies have also revealed changes in CBF perfusion during SCD. In the current study, multi‐delay pCASL, which had good accuracy and consistency was used to measure the cross‐sectional and longitudinal cerebral blood perfusion in the two groups, and the results revealed that increased CBF in the left precuneus at baseline was an independent risk factor for the progression of SCD (Thomas et al. 2021). Hays et al. found that participants with SCD showed higher CBF in the posterior cingulate cortex, middle temporal gyrus, hippocampus, fusiform gyrus, and inferior frontal gyrus (Hays et al. 2018). Another study compared the CBF in the ROIs of 162 participants in the AD neuroimaging program, and the results showed that the CBF of the hippocampus and inferior parietal lobe increased in participants with SCD compared with HCs. Moreover, W. Li et al. (2022) showed that compared with HCs, patients with SCD had increased CBF values in the left hippocampus, left middle temporal gyrus, bilateral posterior cingulate gyrus, and bilateral precuneus. Hyperperfusion of classical cognitive brain areas may indicate early neurovascular dysregulation and early cognitive inefficiency. Therefore, a higher CBF is required to maintain tissue metabolism and cognitive function. At present, longitudinal studies on ASL related to SCD are lacking. The findings of this study suggested that there was compensatory hyperperfusion in the left precuneus in participants with SCD, which might be related to the precuneus as the main hub of the default mode network prone to Aβ pathological deposition in the early stage of the disease (Cui et al. 2025). X. Y. Li et al. (2024) further verified this view using resting‐state fMRI, revealing that the functional connectivity and local consistency of precuneus in the Aβ‐positive group were higher than those in the Aβ‐negative group in participants with SCD, suggesting that the potential mechanism of increased functional connectivity and metabolic perfusion in precuneus in the early stage of disease is helpful in maintaining normal cognitive function and behavior. It is also believed that hyperperfusion in the early stage of the disease may be related to an increase in neuroinflammation and vasodilation induced by Aβ deposition in the brain region, so further mechanism studies are needed to clarify.

Previous studies have found that SCD also causes brain structural changes, such as atrophy of the hippocampus and medial temporal lobe (Hong et al. 2023). However, consistent with the results of Yang et al. (2023), we found no significant difference in ROIs between HCs and participants with SCD, which may be related to different image data processing methods and ROI selection. However, this further suggests that CBF changes measured by multi‐delay pCASL may be more sensitive and stable as a diagnostic imaging marker for SCD than brain structural changes.

This study has some limitations. First, this study is a single‐center study with a certain degree of selection bias, and the conclusions of the study need to be further verified in a multi‐center, large sample size cohort. Second, it is necessary to further expand the sample size and combine pathological markers in cerebrospinal fluid and blood to establish a more stable multi‐omics SCD diagnosis and disease prediction model for accurate early diagnosis and intervention in AD and related cognitive impairment. Third, the follow‐up loss rate in this study was high, and the follow‐up time was relatively short. AD progression is a prolonged and dynamic process that can span years or even decades, beginning in the preclinical stage long before the onset of overt clinical symptoms. Therefore, it is necessary to continue to expand the follow‐up period in future studies to more accurately capture the trajectory of cognitive decline and to validate the long‐term predictive utility of the identified biomarkers.

## 5 Conclusion

In conclusion, through the analysis of clinical and imaging data of participants with SCD at baseline and follow‐up, our results revealed that poor sleep quality was an independent risk factor for SCD progression and emphasized the importance of improving sleep quality to prevent SCD. In this study, pCASL was used for the first time to longitudinally evaluate the outcomes of participants with SCD. The results showed that the compensatory increase in precuneus perfusion at baseline was an independent risk factor for the progression of SCD. The importance of sleep improvement for early prevention and the potential of left precuneus as a target for intervention are further proposed. Finally, we developed a predictive model with high discrimination and clinical utility that provides convenient, sensitive, and repeatable clinical and imaging markers for early diagnosis, disease monitoring, and treatment of SCD.

## Author Contributions

**Wenyi Li**: conceptualization, formal analysis, investigation, methodology, writing – original draft. **Jiwei Jiang**: conceptualization, methodology, data curation, writing – review and editing, project administration. **Qiwei Ren**: investigation. **Min Zhao**: investigation. **Linlin Wang**: investigation. **Shiyi Yang**: investigation. **Shirui Jiang**: investigation. **Tianlin Jiang**: investigation. **Huiying Zhang**: investigation. **Jun Xu**: writing – review and editing, supervision, project administration.

## Ethics Statement

This research was approved by the Institutional Review Board of the Beijing Tiantan Hospital of Capital Medical University (KY‐2021‐028‐01).

## Conflicts of Interest

The authors declare no conflicts of interest.

## Peer Review

The peer review history for this article is available at https://publons.com/publon/10.1002/brb3.70719.

## Supporting information




**Supplementary Materials**: brb370719‐sup‐0001‐SuppMat.docx

## Data Availability

The data will be made available from the authors upon reasonable request.
