# Supplementary material for: Development of a Predictive Model for the Progression of Subjective Cognitive Decline: A Longitudinal Study
Source: Brain Behav. 2025 Aug 12;15(8):e70719. doi: 10.1002/brb3.70719 (PMC12340233; doi:10.1002/brb3.70719)
Supplement: Supplementary file 1 — Supplementary Materials: brb370719‐sup‐0001‐SuppMat.docx [file BRB3-15-e70719-s001.docx]

**Development of a predictive model for the progression of subjective cognitive decline: a longitudinal study**


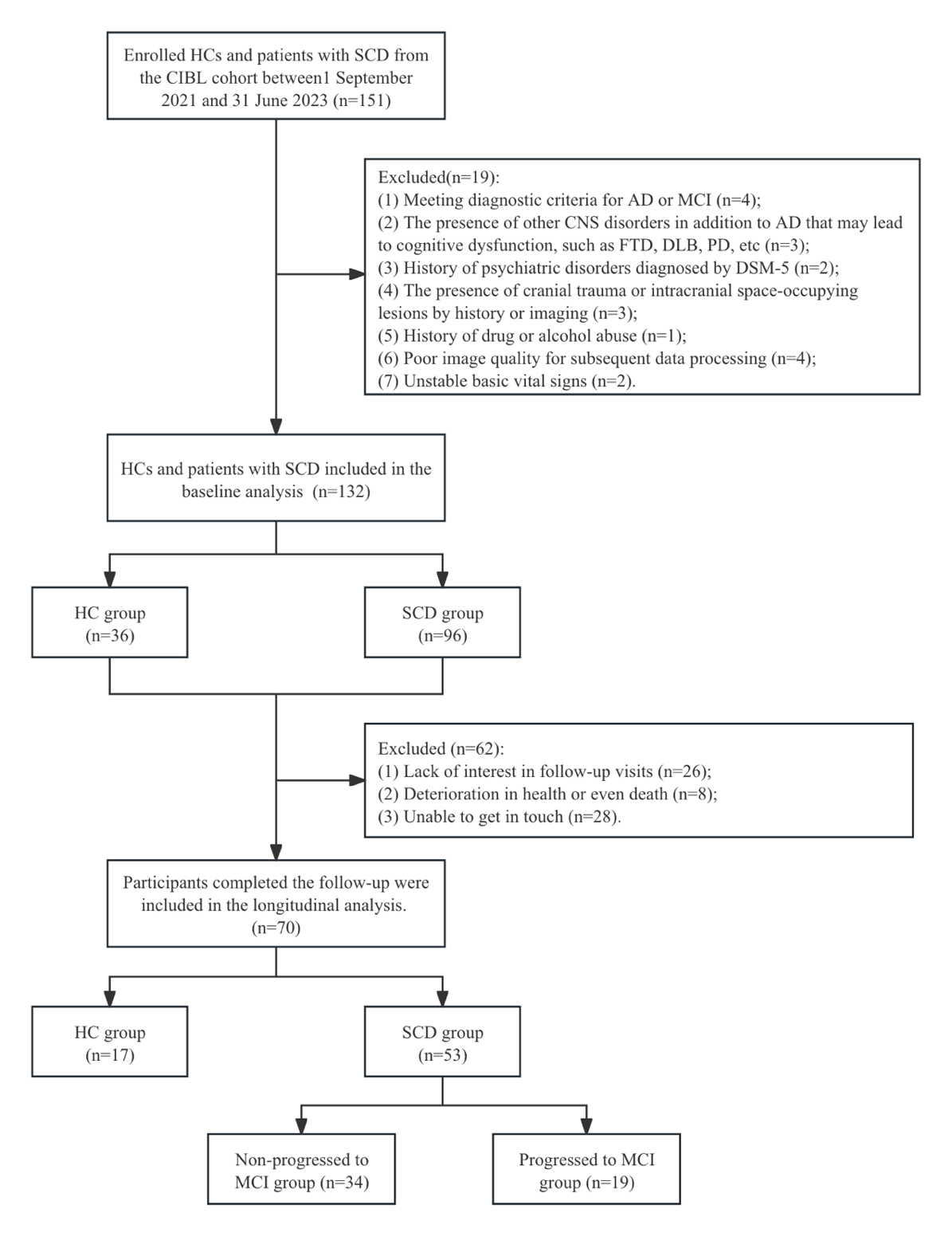


**Supplement Figure 1.** **A flow chart of participants selection and data analysis**.

Abbreviations: HC, HC, healthy control; SCD, subjective cognitive decline; AD, Alzheimer’s disease; MCI, mild cognitive impairment; CNS, central nervous system; FTD, Frontotemporal dementia; DLB, dementia with Lewy bodies; PD, Parkinson's disease; DSM-5 Diagnostic and Statistical Manual of Mental Disorders, 5th Edition.

**Supplementary material 01. Comprehensive and detailed neuropsychological assessments**

The Beijing version of the Montreal Cognitive Assessment (MoCA) was used to evaluate global cognitive function. Cognitive impairment was defined according to education-adjusted cutoff scores: a total MoCA score ≤24 for individuals with more than 6 years of education, ≤20 for those with 1 to 6 years of education, and ≤14 for those with no formal education.^1^

The Activities of Daily Living (ADL) scale was employed to assess basic daily functioning and instrumental abilities. It comprises 20 items, each rated on a 1–4 scale, yielding a total score ranging from 20 to 80, with higher scores indicating more severe functional impairment.^2^

The Neuropsychiatric Inventory (NPI) was used to evaluate neuropsychiatric symptoms. It assesses 12 domains using a 3-point scale for severity and a 4-point scale for frequency. The total NPI score was calculated by summing the individual domain scores.^3^

The Pittsburgh Sleep Quality Index (PSQI) was administered to assess sleep quality over the past month. The PSQI consists of 19 items grouped into seven components, each scored from 0 to 3. The total PSQI score is the sum of the component scores, with higher scores reflecting poorer sleep quality.^4^

**Supplement Table 1. Baseline** **characteristics of participants with follow-up and lost follow-up**

|  | Follow-up group (n=70) | Lost follow-up(n=62) | t/ꭓ^2^/z | *P* value |
| --- | --- | --- | --- | --- |
| Age of enrollment (mean$\pm$SD, years) | 62.34±7.46 | 61.69±7.97 | 0.022 | 0.637 |
| Sex [female, (%)] | 49 (70.00) | 50 (80.65) | 1.987 | 0.159 |
| Education (median [IQR], years) | 12.00(9.00, 16.00) | 13.50 (12.50, 16.00) | -1.371 | 0.170 |
| *APOE*ε4 carrier [Yes, n (%)] | 12 (17.14) | 11 (17.74) | 0.008 | 0.928 |
| BMI (mean$\pm$SD, kg/m^2^) | 24.00±2.32 | 24.13±3.12 | 4.710 | 0.777 |
| Hypertension [Yes, n (%)] | 27 (38.57) | 18 (29.03) | 1.332 | 0.249 |
| Diabetes [Yes, n (%)] | 10 (14.29) | 9 (14.52） | 0.001 | 0.970 |
| Hyperlipidemia [Yes, n (%)] | 23 (32.86) | 27 (43.55) | 1.597 | 0.206 |
| Stroke/TIA [Yes, n (%)] | 6 (8.57) | 3 (4.83) | — | 0.500 |
| Smoking [Yes, n (%)] | 17 (24.29) | 9 (14.52) | 1.984 | 0.159 |
| Drinking [Yes, n (%)] | 7 (10.00) | 6 (9.68) | 0.421 | 0.521 |
| Family history of dementia [Yes, n (%)] | 18 (25.71) | 16 (25.81) | 0.380 | 0.538 |
| MoCA (median [IQR], scores) | 27.00 (26.00, 28.00) | 27.00 (26.00, 28.00) | -0.369 | 0.712 |
| ADL (median [IQR], scores) | 20.00 (20.00, 20.00) | 20.00 (20.00, 20.00) | -0.477 | 0.633 |
| NPI (median [IQR], scores) | 0.00 (0.00, 1.00) | 0.00 (0.00, 1.00) | -0.430 | 0.667 |
| PSQI (median [IQR], scores) | 7.00 (4.00, 10.00) | 5.00 (3.25, 9.00) | -1.004 | 0.315 |

Abbreviations: —, *P* values were evaluated using Fisher exact tests. HC, healthy control; SCD, subjective cognitive decline; BMI, body mass index; *APOE*ε4, apolipoprotein E epsilon 4; MoCA, Montreal cognitive assessment; ADL, Activities of Daily Living; NPI, Neuropsychiatric Inventory; PSQI, Pittsburgh sleep quality index.

**Supplement Table 2. The follow-up characteristics of HCs and participants with SCD**

|  | HCs (n=17) | SCD (n=53) | t/ꭓ^2^/z | *P* value |
| --- | --- | --- | --- | --- |
| Age of follow-up (mean$\pm$SD, years) | 67.50±7.01 | 65.60±6.45 | 0.671 | 0.505 |
| Sex [female, (%)] | 11 (64.71) | 33 (62.26) | 0.176 | 0.675 |
| Follow-up time (median [IQR], months) | 12.00 (11.75, 13.00) | 12.00 (10.00, 13.00) | -0.987 | 0.324 |
| Progressed to MCI [Yes, n (%)] | 2 (11.76) | 19 (35.85) | — | 0.045 |

Abbreviations: —, *P* values were evaluated using Fisher exact tests. HC, healthy control; SCD, subjective cognitive decline; BMI, body mass index; *APOE*ε4, apolipoprotein E epsilon 4; MoCA, Montreal cognitive assessment; ADL, Activities of Daily Living; NPI, Neuropsychiatric Inventory; PSQI, Pittsburgh sleep quality index.

**References**

1. Yu J, Li J, Huang X. The Beijing version of the Montreal Cognitive Assessment as a brief screening tool for mild cognitive impairment: a community-based study. *BMC Psychiatry*. 2012;12:156.

2. Lawton MP, Brody EM. Assessment of older people: self-maintaining and instrumental activities of daily living. Gerontologist. 1969;9(3):179-186.

3. Leung VP, Lam LC, Chiu HF, et al. Validation study of the Chinese version of the neuropsychiatric inventory (CNPI). *Int J Geriatr Psychiatry*. 2001;16(8):789-93.

4. Buysse DJ, Reynolds CF 3rd, Monk TH, et al. The Pittsburgh Sleep Quality Index: a new instrument for psychiatric practice and research. *Psychiatry Res.* 1989;28(2):193-213.
